# Oral Glycine and Sodium Thiosulfate for Lethal Cyanide Ingestion

**DOI:** 10.4172/2167-7972.1000355

**Published:** 2017-06-27

**Authors:** Matthew Brenner, Sarah M Azer, Kyung-Jin Oh, Chang Hoon Han, Jangwoen Lee, Sari B Mahon, Xiaohua Du, David Mukai, Tanya Burney, Mayer Saidian, Adriano Chan, Derek I Straker, Vikhyat S Bebarta, Gerry R Boss

**Affiliations:** 1 Beckman Laser Institute, University of California, Irvine, California, USA; 2 Division of Pulmonary and Critical Care Medicine, Department of Medicine, University of California, Irvine, California, USA; 3 Department of Urology, Chonnam National University Medical School, South Korea; 4 Department of Internal Medicine, National Health Insurance Service Ilsan Hospital, Goyang-si, Geonggi-do, South Korea; 5 Pulmonary Department, The First Affiliated Hospital of Kunming Medical University, Kunming, Yunnan, China; 6 The Institute for Drug Research, School of Pharmacy, Hebrew University of Jerusalem, Jerusalem Israel; 7 Department of Medicine, University of California, San Diego, La Jolla, CA, USA; 8 Department of Emergency Medicine, University of Colorado School of Medicine, Aurora, CO, USA

**Keywords:** Oral cyanide poisoning, Sodium thiosulfate, Gastric alkalization, Continuous wave near infrared spectroscopy, Glycine

## Abstract

**Objective:**

Accidental or intentional cyanide ingestion is an-ever present danger. Rapidly acting, safe, inexpensive oral cyanide antidotes are needed that can neutralize large gastrointestinal cyanide reservoirs. Since humans cannot be exposed to cyanide experimentally, we studied oral cyanide poisoning in rabbits, testing oral sodium thiosulfate with and without gastric alkalization.

**Setting:**

University research laboratory.

**Subjects:**

New Zealand white rabbits.

**Interventions:**

Seven animal groups studied; Groups 1–5 received high dose oral NaCN (50 mg, >LD100) and were treated immediately with oral (*via* nasogastric tube): 1) saline, 2) glycine, 3) sodium thiosulfate or 4) sodium thiosulfate and glycine, or 5) after 2 min with intramuscular injection of sodium nitrite and sodium thiosulfate plus oral sodium thiosulfate and glycine. Groups 6–7 received moderate dose oral NaCN (25 mg, LD70) and delayed intramuscular 6) saline or 7) sodium nitrite-sodium thiosulfate.

**Measurements and Main Results:**

All animals in the high dose NaCN group receiving oral saline or glycine died very rapidly, with a trend towards delayed death in glycine-treated animals; saline *versus* glycine-treated animals died at 10.3+3.9 and 14.6+5.9 min, respectively (p=0.13). In contrast, all sodium thiosulfate-treated high dose cyanide animals survived (p<0.01), with more rapid recovery in animals receiving both thiosulfate and glycine, compared to thiosulfate alone (p<0.03). Delayed intramuscular treatment alone in the moderate cyanide dose animals increased survival over control animals from 30% to 71%. Delayed treatment in high dose cyanide animals was not as effective as immediate treatment, but did increase survival time and rescued 29% of animals (p<0.01 *versus* cyanide alone).

**Conclusions:**

Oral sodium thiosulfate with gastric alkalization rescued animals from lethal doses of ingested cyanide. The combination of oral glycine and sodium thiosulfate may have potential for treating high dose acute cyanide ingestion and merits further investigation. The combination of systemic and oral therapy may provide further options.

## Introduction

Cyanide is highly toxic, and exposures can result from accidental or intentional causes, including inhalation or ingestion from industrial processes, chemical weapons, terrorism, or suicidal acts [1–5]. Acute and chronic dietary cyanide ingestion occurs in third-world countries where consumption of unprocessed crops, including cassava, leads to significant oral cyanide exposure [6].

Regardless of exposure method, death from acute cyanide poisoning is usually rapid, within minutes from inhalation, and within minutes to hours following oral ingestion [7]. A major mechanism of cyanide toxicity is inhibition of cytochrome c oxidase, a key component of complex IV of the mitochondrial electron transport system. This induces cellular hypoxia, leading to seizures, respiratory depression, cardiac arrhythmias, and cardiovascular collapse, with characteristic elevation of plasma lactate and venous oxygen concentrations [4].

Acute toxicity from oral cyanide ingestion is dose dependent, but other factors such as solubility of the ingested cyanide or cyanogenic compound and presence of food in the gastrointestinal tract can affect clinical severity and time until death [4]. The current recommendation for treating acute oral cyanide poisoning is to neutralize absorbed cyanide by intravenously administered drugs such as hydroxocobalamin, nitrites, and sodium thiosulfate, in addition to supportive care. No treatment exists for preventing absorption of the large gastrointestinal reservoir of cyanide that may be present following ingestion. Historical treatments for acutely ingested poisons included induction of vomiting and/or gastrointestinal lavage, with limited effectiveness. Inefficiency of gastric emptying, rapid onset of seizures, loss of consciousness, and aspiration risks has led to abandoning these approaches for oral cyanide poising. Thus, treatments that directly neutralize ingested cyanide within the gastrointestinal (GI) tract are needed.

Sodium thiosulfate acts as a sulfur donor for the enzyme rhodanese, which transfers the sulfur to cyanide generating thiocyanate, a relatively non-toxic product [5,8–10]. At high concentrations, thiosulfate is absorbed from the GI tract [11,12], and, under certain conditions, thiosulfate can react non-enzymatically with cyanide to generate thiocyanate [13,14]. Thus, oral/enteral thiosulfate could potentially neutralize cyanide in the GI tract, as well as metabolize systemically absorbed cyanide. Since sodium thiosulfate is inexpensive, and has minimal toxicity [15], large quantities could be administered enterally.

On exposure to acidic gastric conditions, ingested cyanide ion is rapidly converted to hydrogen cyanide gas due to its pKa of 9.3 and boiling point of 26.3°C. Thus, cyanide is likely absorbed as hydrogen cyanide gas, rather than as cyanide ion [16], and cyanide gas in gastric or intestinal bubbles (as opposed to dissolved gas) may be less accessible to neutralizing antidotes within the GI tract. We hypothesized that raising gastric pH with an orally administered buffer should maintain cyanide primarily as cyanide ion, thus delaying systemic absorption. At a pH above 10.3, >90% of ingested cyanide would be in the ionized form. We recently showed we could rescue rabbits from a lethal dose of oral cyanide using the combination of oral cobinamide and sodium carbonate [17]. However, cobinamide is relatively expensive, is absorbed from the GI tract and has some toxicity when administered parenterally at high doses, and is not FDA approved. Furthermore, while sodium carbonate is an effective and safe buffer, large volumes of CO_2_ gas form when added to the acidic stomach. This creates a large gas “headspace” where hydrogen cyanide gas may not be as accessible for neutralization. Glycine is also an effective buffer at high pH, but does not generate gas on exposure to acid. Human ingestion of up to 31 g of glycine per day is without serious side effects [18,19]. Thus, we proposed that glycine would be an ideal agent to raise gastric pH to delay cyanide absorption.

Our primary aim is to develop safe and inexpensive methods for treating oral cyanide poisoning. For developing cyanide antidotes, animal studies are equivalent to human clinical trials, since efficacy cannot be tested in humans, and FDA approval is based solely on animal experiments. Some victims of oral cyanide ingestion may be asymptomatic shortly following ingestion, or it may be unknown if ingestion actually occurred, particularly in mass exposure situations. For asymptomatic patients or unexposed persons who think they were exposed, treatment must be safe and inexpensive and preferably by the oral route. Patients who are unconscious or severely symptomatic may require combined systemic and enteral antidote treatment. To investigate this range of possible exposure and severity scenarios, we used lethal rabbit models of moderate and high dose oral cyanide ingestion, assessing survival, physiologic parameters, and metabolic effects of cyanide; the latter were monitored using continuous wave near infrared spectroscopy [20–25]. We found that high dose oral thiosulfate is effective in lethal oral cyanide poisoning.

## Materials and Methods

### Animal preparation

Pathogen free New Zealand White rabbits from 3.5 to 4.5 g were used. All procedures were reviewed and approved by the Institutional Animal Care Committee of the University of California, Irvine. Each rabbit was deprived of food for at least 16 h and water for 3 h prior to the experiment. An Elizabethan collar was placed to prevent coprophagy during the 16 h pre-treatment period.

Animals were anesthetized with a 2: 1 Ketamine HCl (100 mg/ml, Ketaject, Phoenix Pharmaceutical Inc., St. Joseph, MI): Xylazine (20 mg/ml, Anased, Lloyd Laboratories, Shenandoah, IA) intramuscular injection. A 23 gauge 1-inch catheter was then inserted in the marginal ear vein for intravenous access. The animals were intubated using a 3.5 mm cuffed endotracheal tube, which was connected to a Bickford non-rebreathing circuit. Animals continuously inhaled a mixture of 1.5–2.5% isoflurane and room air through an Ohmeda V.M.C anesthesia machine. A 14 French naso-tracheal suctioning catheter connected to a three-way stopcock was introduced through the mouth into the stomach. Position was confirmed by auscultation of injected air and withdrawal of gastric fluid contents. Heart rate and oxygen saturation (SpO_2_) were monitored through a pulse oximeter (Biox 3700 Pulse Oximeter, Ohmeda, Boulder, CO) ear probe (Datex-Ohmeda TS-E4-H) placed on the animal’s cheek. Respiratory rate, end tidal CO_2_, and end tidal O_2_ were monitored through a Datex Ohmeda, General Electric, S/5 Patient Monitor connected to the endotracheal tube. The femoral artery and vein in the left groin were isolated by blunt dissection, and catheterized with 12 inch, 18 g catheters (C-PMA-400-FA, Cook Inc, Bloomington, IN), with three-way stopcocks connected to each catheter. Blood pressure was measured by a calibrated pressure transducer (TSD104A Transducer and MP100 WSW System Biopac Systems, Inc., Santa Barbara, CA) connected to the arterial line.

### Gastric fluid sampling

Gastric fluid was sampled at baseline, after sodium thiosulfate and glycine administration, and at 2.5, 7.5, 15, 30, 45, and 60 min post sodium cyanide installation.

### Blood sampling and data Collection

Cyanide analysis, blood gasses, SpO_2_, and metabolic data were obtained from blood samples collected at baseline, at the time of cyanide administration, and at 2.5, 5, 10, 15, 30, 45, and 60 min post cyanide administration. Blood pressure was monitored continuously and recorded every minute for the first 10 min after cyanide installation, then every 15 min thereafter.

Animals that survived until 60 min post cyanide administration were considered to have survived and were euthanized with 1 cc of Euthasol (390 mg Pentobarbital Sodium/50 mg Phenytoin Sodium) (Euthasol, Virbac AH, Inc., Fort Worth, Texas) administered through the marginal ear vein. Animals that died before 60 min were considered “non-survivors.”

### Antidotes and reagents

For use in moderate and high dose cyanide experiments, 25 or 50 mg NaCN (Sigma-Aldrich), respectively, was dissolved in 10 ml of 0.9% saline (Revival Animal Health). Glycine (2 M, Sigma-Aldrich) was adjusted to pH 11 with 10 N NaOH, and 5 to 12 cc were given through the gastric tube in 2.5 cc increments to adjust stomach pH to >9. Averages of 7.5 cc of glycine were needed to reach the target stomach pH. Sodium thiosulfate pentahydrate (2.5 M, Sigma-Aldrich) was given orally (4 cc in 2 doses given 5 min apart) or by intramuscular injection (0.62 cc). Sodium nitrite (Sigma-Aldrich) was given intramuscularly as 0.32 cc of an 800 mM solution.

### Study design and treatment groups

Rabbits were divided into seven groups of seven animals per group, except the moderate cyanide dose group treated with saline (Group 6), where 10 animals were used. Thus, a total of 52 animals were studied.

Glycine was administered as a 2 M solution adjusted to pH 11 with NaOH; 5–12 cc (average: 7.5cc) were administered to achieve a gastric pH >9. Sodium thiosulfate was administered as two equal instillations of 4 cc of a 2.5 M solution, given five minutes apart after gastric alkalization, in groups 3 and 4. In the high dose delayed treatment Group 5, 30 cc of oral thiosulfate with 7.5 cc 2 M glycine plus simultaneous IM injections of 0.64 cc 2.5 M thiosulfate and 800 mM NaNO were administered after apnea onset or at 2 min post-cyanide exposure, whichever came first. In the moderate dose Group 7, simultaneous IM injections of 0.64 cc 2.5 M thiosulfate and 800 mM NaNO were administered immediately post-cyanide exposure.

The seven groups are as follows:

High Dose Cyanide (50 mg NaCN) with Simultaneous Oral Therapy
Group 1: Saline (control group),
Group 2: Glycine,
Group 3: Sodium thiosulfate,
Group 4: Glycine and sodium thiosulfate,
High Dose Cyanide (50 mg NaCN) with Delayed Oral and Intramuscular Therapy,
Group 5: Oral glycine and thiosulfate plus intramuscular nitrite-thiosulfate given at apnea or 2 minutes post ingestion, whichever came first,
Moderate Dose Cyanide (25 mg NaCN) with Delayed Intramuscular Therapy,
Group 6: Saline (control group),
Group 7: Intramuscular nitrite-thiosulfate given at apnea or 2 min post ingestion, whichever came first

Six of the 10 animals in Group 6 were previously used as controls and reported in a prior study of oral cobinamide treatment [17].

### CWNIRS measurements

The CWNIRS system was designed to optimize detection of a metabolic poison such as cyanide [26]. It consists of a light source (HL 2000, Ocean Optics, FL), a CCD spectrometer (BTC111E, B&WTek, DE), and customized optical fiber guides. It acquires a full spectrum of transmitted light (600~1000 nm) every second and collects light intensity values at five wavelengths (732, 758, 805, 840, 880 nm) to calculate changes in tissue oxyhemoglobin (OHb) and deoxyhemoglobin (RHb) using a modified Beer-Lamberts’ law. The two wavelengths below 805 nm, an isosbestic point, have good sensitivity for detecting deoxyhemoglobin and the two wavelengths above the isosbestic point have good sensitivity for detecting oxyhemoglobin.

CWNIRS recovery times were compared to midpoint return of oxyhemoglobin toward baseline. Rate of return was analyzed by slope analysis from 25 to 75% of recovery from peak to baseline. Maximal change was determined from baseline to peak values.

### Measurement of Red Blood Cell (RBC) cyanide concentration

Cyanide in blood is bound almost exclusively to ferric (met) hemoglobin in RBCs; thus, the blood cyanide concentration can be measured by measuring cyanide in RBCs [27]. Whole blood collected from animals was immediately cooled to 4°C, centrifuged, and the plasma and RBC fractions separated. Samples were kept at 4°C and analyzed within 48 h. The RBCs were lysed in ice-cold water, and the lysates were placed into the outer compartment of a Conway microdiffusion cell. A volume of 10% trichloroacetic acid equal to the lysate volume was also added to the outer compartment, and an alkalized cobinamide solution was added to the center compartment. The cell was capped, and the lysate was mixed with the tricholoracetic acid by gently tilting the chamber. The trichloroacetic acid denatures the hemoglobin and releases hydrogen cyanide gas, which was trapped in the cobinamide solution. The resulting dicyanocobinamide is measured spectrophotometrically as described previously [28]. Cyanide concentrations were determined from standard curves using freshly prepared KCN dissolved in 1 mM NaOH. Sample duplicates showed <15% variation.

### Measurement of plasma and gastric fluid thiocyanate concentration

Thiocyanate in the plasma was reduced to cyanide using potassium permanganate as described previously [29]. The resulting cyanide was measured as described above.

Samples of gastric fluid were withdrawn from the stomach via the nasogastric tube at baseline, after alkalization, and at 2.5, 7.5, 30, and 45 min post cyanide administration. Thiocyanate in the fluid was measured by adding samples to acidic ferric nitrate, which yields a yellow-orange product that can be quantified spectrophotometrically at 460 nm. The thiocyanate concentration was determined from standard curves, with sample duplicates showing < 15% variation.

### Measurement of cyanide reaction with sodium thiosulfate

We assessed reaction rates of sodium thiosulfate with sodium cyanide under conditions simulating the intragastric milieu, both under native conditions and after adding glycine buffer. Thus, we incubated 750 mM sodium thiosulfate with 40 mM NaCN at 37°C. We conducted the experiment at low and high pH (pH 2.0 and 10.0, respectively), and measured thiocyanate production over varying periods of time from 5 to 60 min using the acidic ferric nitrate reagent described above. We compared results to a standard curve of sodium thiocyanate.

### Statistical analysis

Survival time curves were compared by Kaplan Meier survival analysis (Systat Software, San Jose, CA) with significance calculated by log-rank (Mantel-Cox) tests (Prism 6 Software, Graphpad, Inc., La Jolla, CA), and overall survival assessed by a Chi-square analysis between individual groups with adjustment for multiple comparisons. Gastric pH, concentrations of cyanide and metabolites, and recovery rates across groups were compared by ANOVA with repeated measures (Systat Software, San Jose, CA).

### Power analysis

For the primary outcome variable of survival, power analysis yielded 6 animals per group in animals treated with high dose cyanide, if mortality is 100% in control animals and survival is >70% in treated animals, with two tailed p<0.05 and beta error avoidance at >80%. CWNIRS recovery times were compared by time to midpoint return of oxyhemoglobin curves to baseline, and rate of return was analyzed by slope analysis comparison by ANOVA.

## Results

### Measurement of cyanide reaction with sodium thiosulfate

Cyanide can react directly with thiosulfate, generating thiocyanate, but relatively high concentrations of both reactants are needed and the reaction is favored under alkaline conditions [14,30]. We investigated whether the reaction could occur under conditions that simulated those of our *in vivo* experiments. We found that at pH 2, with 750 mM sodium thiosulfate and 40 mM NaCN, the rate of thiocyanate production was 20 nmol/min, whereas at pH 10, thiocyanate production was about eight-fold faster or 155 nmol/min.

### Gastric pH measurements

**Figure 1:**
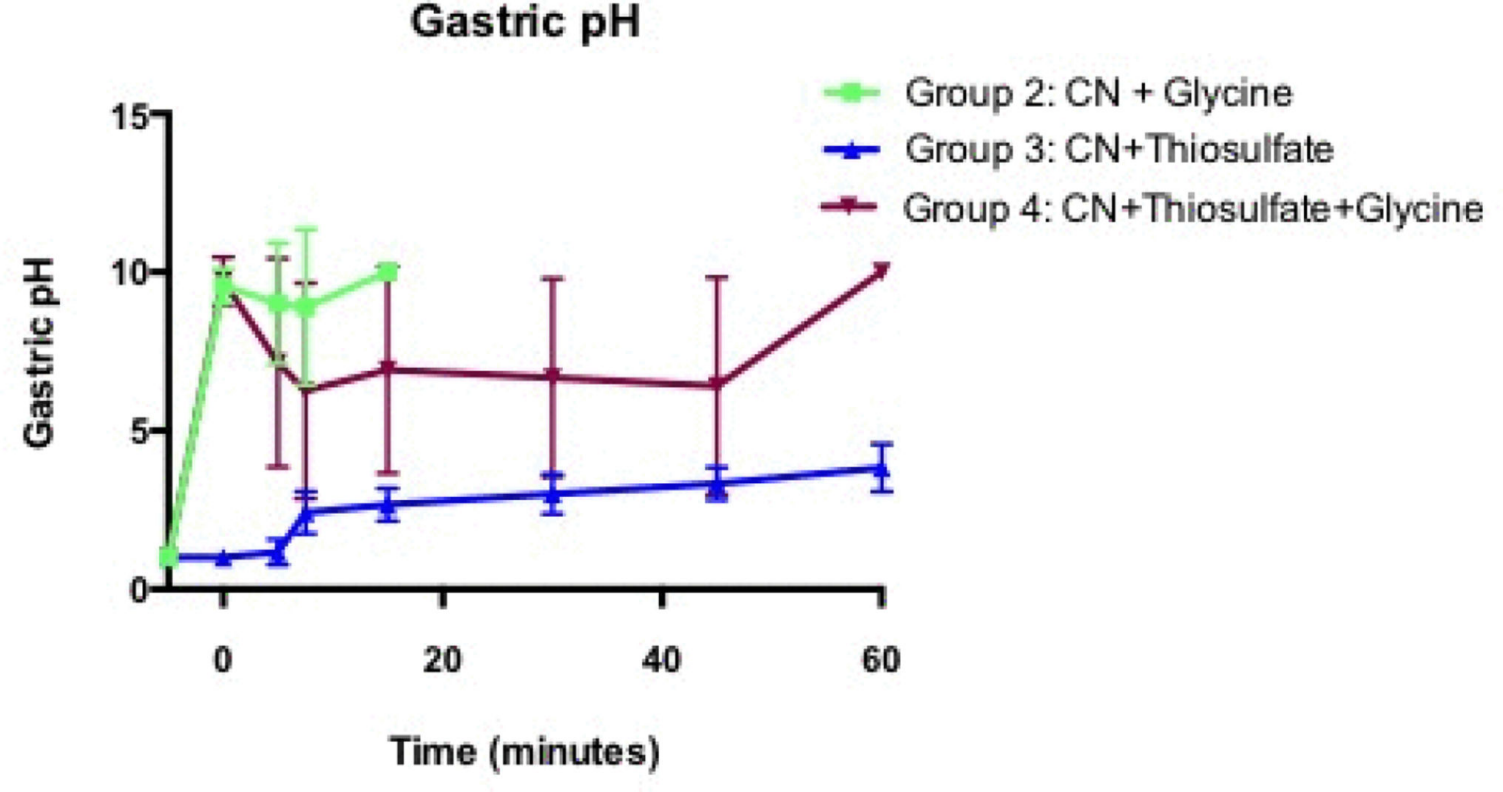
Gastric pH in the high dose cyanide treatment groups. Baseline pH was measured prior to instillation of drugs or cyanide. Time 0 is immediately following instillation of drugs and cyanide. Results are shown for Groups 2, 3, and 4. Group 1 animals (cyanide alone) did not survive long enough for multiple measurements. The increase in pH following glycine administration is significantly different from non-glycine-treated animals p<0.01 by ANOVA.

Gastric pH measurements were performed in the high dose cyanide animals at intervals pre- and post-cyanide exposure (Figure 1). All animals had baseline gastric pH <2. Group 1 animals (cyanide alone) died before serial gastric pH measurements could be made and are not shown in Figure 1.

Group 2 animals that received cyanide and glycine had sustained gastric pH values above 8 until death (mean pH range 9.6–10, following glycine and cyanide). Group 3 animals that received cyanide and sodium thiosulfate had low pH values initially (pH~1) that rose slowly to 3.8+0.3 at 60 min.

Group 4 animals administered glycine plus sodium thiosulfate and cyanide continued to have more alkaline pH in the stomach than group 3 animals, 9.7+0.3 after administration, but did have a gradual decrease in pH to 6.4+1.5 at 60 min (p<0.01 by ANOVA across groups).

### Survival with high dose cyanide exposures

#### Effect of High Dose Oral Cyanide (Group 1)

**Figure 2:**
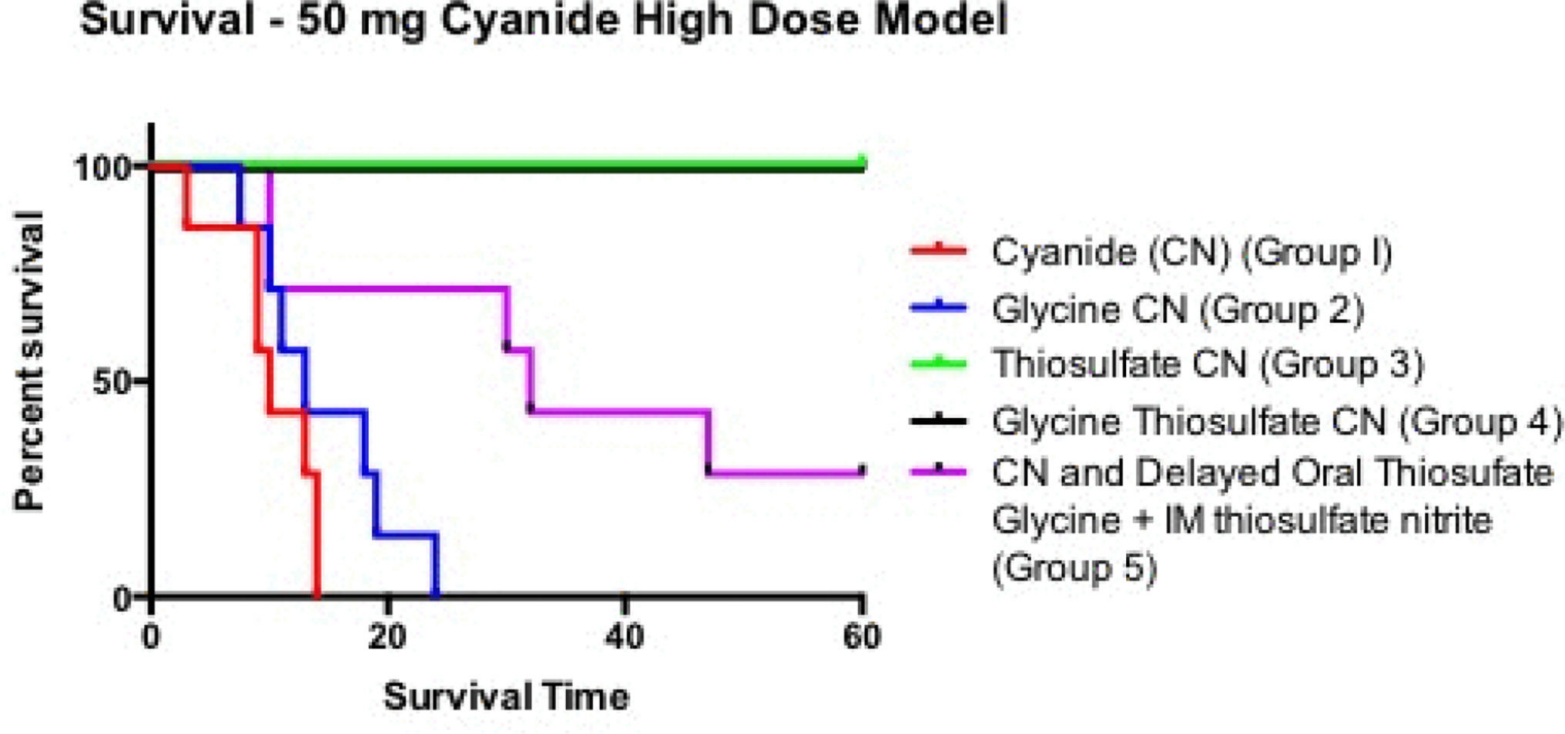
Survival Curves of High Dose Cyanide Groups. Group 1, cyanide alone and Group 2, cyanide plus glycine, animals all died. Group 3 animals receiving oral thiosulfate survived the full 60 min, as did Group 4 animals receiving glycine and sodium thiosulfate (compared to Group 1 and 2 animals, p<0.01 by Log-rank analysis). Of Group 5 animals receiving high dose cyanide followed by delayed intramuscular thiosulfate nitrite and oral thiosulfate plus glycine, 29% survived (p<0.01 compared to Group 1 and 2 animals).

Animals in Group 1, receiving saline with high dose cyanide, survived an average of 10.3 ± 3.9 min (range 3 to 14 min, 95% CI 6.7 to 13.9) (Figure 2). All animals in Group 1 became apneic between 2 and 3.5 min post cyanide ingestion (mean 2.6+0.7 min, 95% CI 2.0–3.3 min). Thus, the model is rapidly and uniformly lethal (Figure 2).

#### Effect of Glycine (Group 2)

Animals in Group 2, receiving glycine buffer with high dose cyanide, survived an average 14.6 ± 5.9 min (range 7.5–24, 95% CI 9.2 to 20.1 min) (Figure 2). Thus gastric alkalization prolonged time to death by ~40% (p=0.13 compared to Group 1 animals), but did not prevent death.

#### Effect of Sodium Thiosulfate (Group 3)

Animals in Group 3, receiving oral sodium thiosulfate, recovered slowly as demonstrated by CWNIRS monitoring of oxy- and deoxyhemoglobin (see below), but all survived the 60 min experimental period (Figure 2; p<0.01 for survival compared to Groups 1 and 2).

#### Effect of Glycine and Sodium Thiosulfate (Group 4)

Animals in Group 4, receiving glycine buffer and sodium thiosulfate, recovered quickly and survived the full 60 min (Figure 2); p<0.01 for survival compared to Groups 1 and 2).

#### Delayed Combined Oral and Intramuscular Antidote Treatment (Group 5)

Group 5 animals were not treated until onset of apnea or at 2 min post-cyanide, when they were severely compromised clinically. Two of seven animals (29%) survived (Figure 2), and the median survival time increased from 10 to 32 min (Figure 2; p<0.01 for survival compared to Groups 1 and 2).

### Blood cyanide and thiocyanate analysis

**Figure 3:**
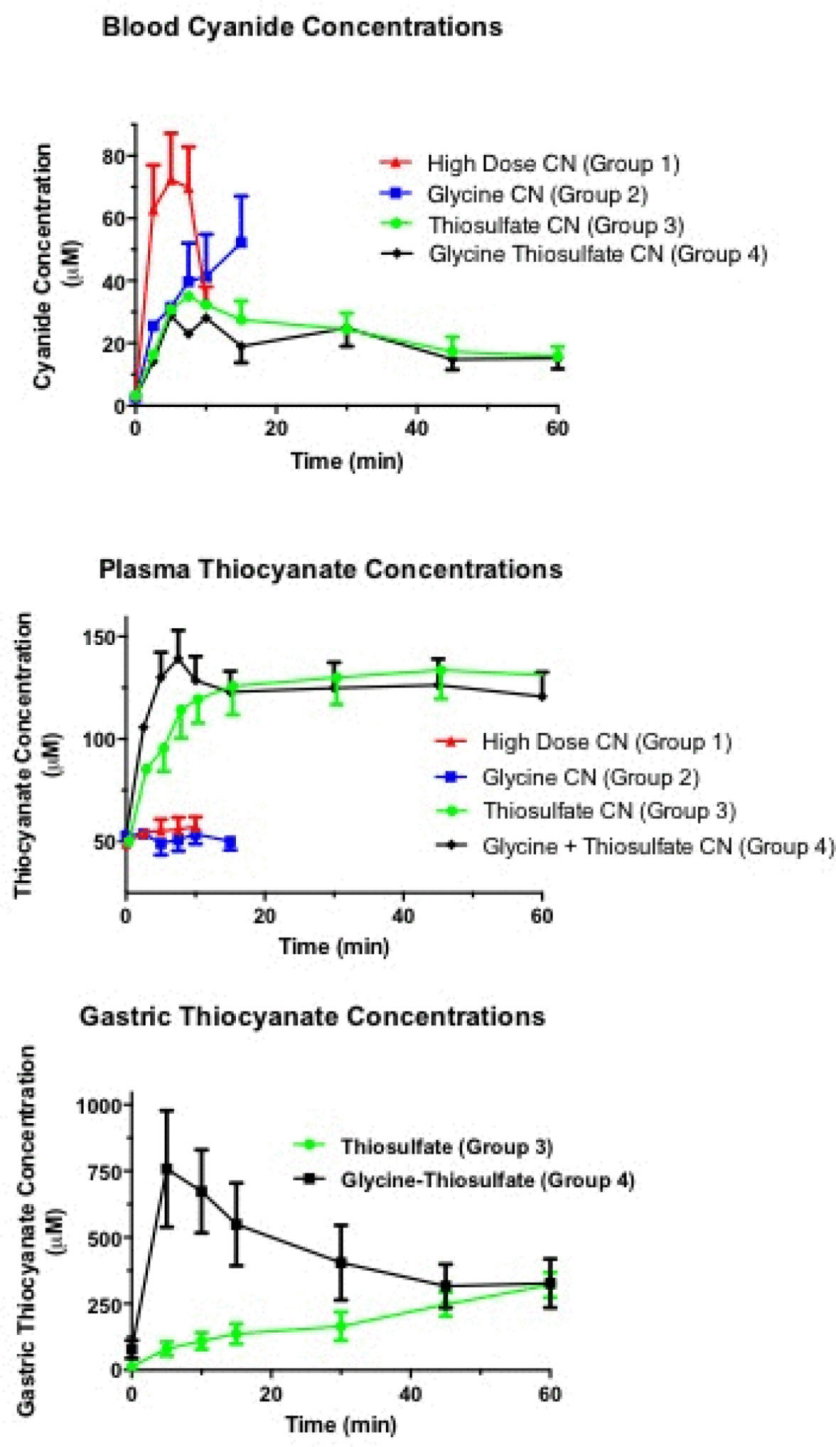
Blood, plasma and gastric cyanide and thiocyanate concentrations: The blood cyanide concentration rose rapidly in control animals (Group 1 cyanide alone), and all expired by 15 min. In Group 2 animals (cyanide plus glycine), the rate of cyanide absorption was significantly lower, but continued to climb until death. In Group 3 and 4 animals receiving sodium thiosulfate or sodium thiosulfate plus glycine, the cyanide concentration rose at the same rate as glycine alone (Group 2 animals), but leveled off and began to decrease by 10 min post ingestion.

All animals had similar, low baseline blood cyanide concentrations (mean 2.9 μM, range 2.6 to 3.1 μM) (Figure 3 top), and plasma thiocyanate concentrations (mean 52.1 μM, range 49.3 to 53.6 μM) (Figure 3 middle).

Group 1 cyanide alone animals demonstrated a rapid increase in the blood cyanide concentration, which peaked at 72+15 μM at 5 min post cyanide instillation (Figure 3 top). In contrast to the large rise in cyanide concentrations, Group 1 animals exhibited only a small rise in plasma thiocyanate concentrations, peaking at 57.2+4.7 μM at 10 min post exposure (Figure 3 middle), consistent with minimal conversion of cyanide to thiocyanate in this group.

Group 2 animals, receiving glycine and cyanide, exhibited a slower rise in blood cyanide concentrations compared to Group 1 animals, yielding 31.5+6 μM at 5 min, but the cyanide concentration continued to rise until expiration, with a mean concentration of 52.2+15 at 15 min (Figure 3 top). Similar to Group 1 animals, Group 2 animals showed no significant change in thiocyanate concentrations, yielding 53.6+4.8 μM at 10 min (Figure 3 middle) (p=NS compared to CN controls), again consistent with limited conversion of cyanide to thiocyanate.

Group 3 animals, receiving sodium thiosulfate, and group Group 4 animals, receiving both glycine and sodium thiosulfate, exhibited a similar initial rise in the blood cyanide concentration. In both these groups, the blood cyanide concentration peaked by 15 min and began to fall subsequently. The blood cyanide concentration was statistically lower in Group 3 and 4 animals compared to Group 1 animals (p<0.01).

The thiocyanate concentration in Group 3 and 4 animals increased at a rate significantly greater than that of Group 1 and 2 animals, consistent with increased conversion of cyanide to thiocyanate in these groups. During the first 10 min following cyanide ingestion (corresponding to when rapid clinical deterioration occurs in control animals), the plasma thiocyanate concentration rose significantly faster in Group 4 than Group 3 animals, pointing to a beneficial effect of the glycine: at baseline, Group 3 and 4 animals had equivalent plasma thiocyanate concentrations [50.1+3.1 (95% CI 42 to 58) and 53.6+4.2 μM (95% CI 48 to 59), respectively], but at 5 min, corresponding concentrations were 95.6+8.0 (95% CI 76 to 116) and 130.0+11.1 μM (95% CI 116 to 144), and at 10 min, they were 119+7 (95% CI 101 to 138) and 129+10 μM (95% CI 116 to 141) (p<0.02 ANOVA with repeated measures).

### Gastric thiocyanate

We measured the gastric fluid thiocyanate concentration in Group 3 and 4 animals, and found it rose very rapidly in the Group 4 animals from 78+59 at baseline to 758+219 μM by 5 min post ingestion (Figure 3 bottom). This is in contrast to a smaller increase in the Group 3 animals, from 13+7μM at baseline to 80+53 μM at 5 min (p<0.05 ANOVA with repeated measures comparing Group 4 to Group 3), demonstrating greater gastric conversion of cyanide to thiocyanate in alkaline conditions *in-vivo*.

### Rate of recovery in surviving animal groups - CWNIRS analysis

We continuously monitored oxygenated hemoglobin (OxyHb) and deoxygenated hemoglobin (DeoxyHb) by CWNIRS over the brain region. In Group 1 and 2 animals, the oxy hemoglobin concentration rose initially for a brief period, then decreased rapidly as the animals expired from cyanide poisoning. The deoxy hemoglobin concentration also rose briefly, but then fell in the terminal phases and tissue blood voume decreased (serial data not shown due to rapid death).

Base excess graph showing changes beginning immediately following ingestion of cyanide in four rabbit groups. Group 1 rabbits administered cyanide alone died rapidly, before 15 min blood gas measurements could be obtained. In Group 2 animals receiving glycine and cyanide, rapid decrease in base excess developed prior to death. Group 3 animals administered sodium thiosulfate with cyanide, showed a rapid sustained decrease in base excess. Group 4 animals that received both sodium thiosulfate and glycine had a gradual mild decrease in base excess following cyanide ingestion (p<0.03 compared to Group 3, ANOVA with repeated measures).

**Figure 4:**
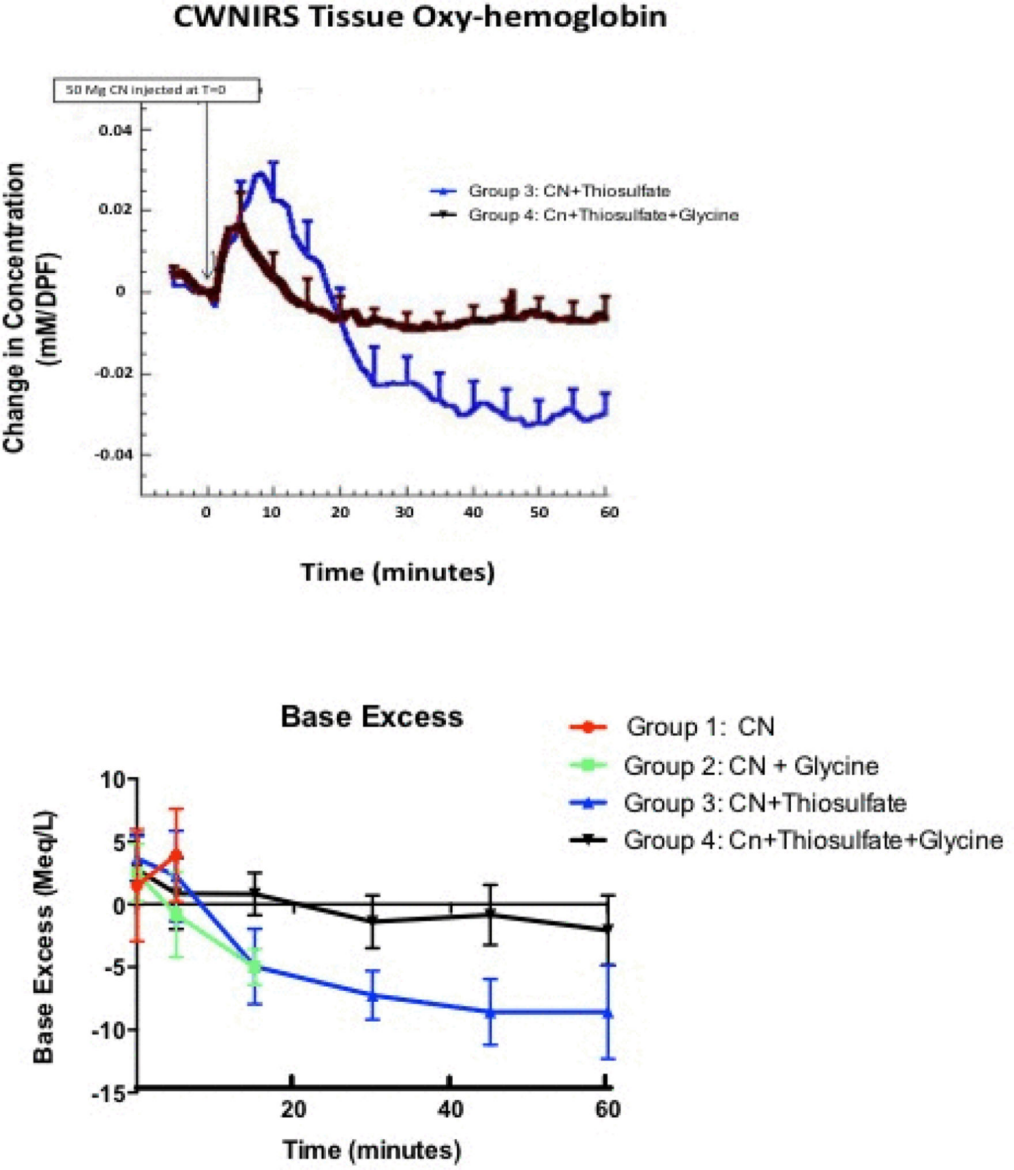
Rate of recovery–CWNIRS and Base Excess, CWNIRS composite curves show changes in brain oxyhemoglobin concentration immediately following ingestion of cyanide in the rabbit groups that recovered. Group 3 animals that received sodium thiosulfate with cyanide had a slower recovery time to baseline than Group 4 animals that received both sodium thiosulfate and glycine (p<0.05).

In Group 3 and 4 animals, the OxyHb concentration rose initially as cyanide was absorbed and animals became progressively unable to extract oxygen from the tissues, but it returned relatively quickly to baseline values in the Group 4 animals. The time to recovery was significantly faster in Group 4 animals averaging 2.4+5.4 min, compared to group 3 animals 17.1+1.9 min (p<0.05) (Figure 4, top).

### Base excess

Base excess is reduced quickly in this model due to cyanide-induced lactic acid production. Base excess was equivalent in all groups at baseline (mean 2.6+3.0 mmol/L, 95% CI 1.4 to 3.1 mmol/L; p>0.7 across groups; Figure 4, bottom). Base excess rapidly decreased in Group 2 animals to (−)5+0.9 mmol/l (95% CI: (−)3.6 to (−)6.4 mmol/L) at 15 min (p<0.01 compared to baseline). Group 3 animals also showed a significant decrease in base excess to (−) 4.9+3.2 mmol/L (95% CI (−)1 to (−9) mmol/L) by 15 min, with a further progressive decrease over the 60 min study period to (−)9.6+2.9 mmol/L (95% CI (−)5 to (−)11) (p<001 compared to baseline). In contrast, in Group 4 animals, base excess decreased at a more gradual rate to 0.9+2.6 mmol/L (95% CI: 1.9 to (−)3.6 mmol/L) at 45 min, and (−)2.7+3.0 mmol/L (95% CI: 07 to (−)4.9 mmol/L) at 60 min (Figure 4) (p=0.01 compared to group 3 animals ANOVA with repeated measures).

### Blood pressure

Baseline blood pressures were similar in all groups, with a mean arterial pressure (MAP) of 45+10 mmHg (95% CI 41–48). The MAP did not differ significantly between Group 3 and Group 4 animals at any time point, gradually decreasing to 31+8 mmHg (95% CI 30–58 mmHg) and 36+10 mmHg (95% CI 26–45 mmHg) at 60 min in Group 3 and 4 animals, respectively (p>0.5).

### Delayed treatment in moderate cyanide dose poisoning

In order to investigate delayed antidote administration and intramuscular administration, the efficacy of intramuscular administration alone was studied in animals using lower dose oral cyanide ingestions (since intramuscular injection alone was previously found not to rescue animals receiving 50 mg of cyanide).

**Figure 5:**
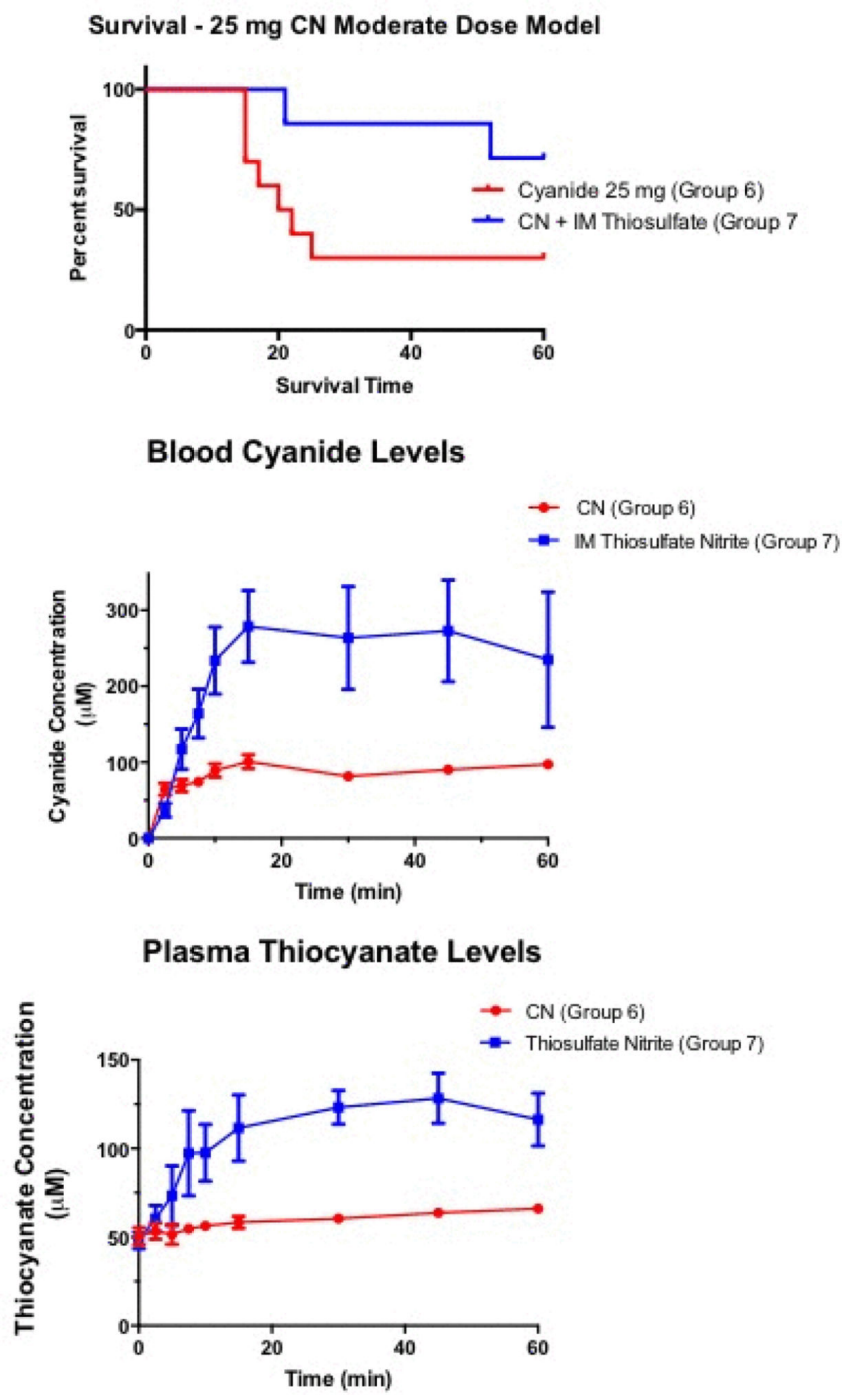
25 mg intramuscular treatment model. Survival: Survival time curves following moderate dose ingested cyanide. Cyanide 25 mg alone (group 6) had 30% survival. Group 7 animals administered ingested cyanide plus intramuscular thiosulfate nitrite had 72% overall survival (p<0.05).

Thirty percent of Group 6 animals that received 25 mg of cyanide and were treated with intramuscular saline placebo survived, with a median survival time of 21 min (compared to 10 min for Group 1 animals that received 50 mg cyanide) (Figure 5, p<0.001, Log-rank for difference between the Group 6 and Group 1). Of Group 7 animals that received delayed intramuscular injection of sodium nitrite and sodium thiosulfate after CN ingestion, 71% survived (p<0.05 compared to Group 6 animals; Figure 5).

Blood CN Concentration: easured blood CN levels rose higher in animals receiving intramuscular thiosulfate thiosulfate/sodium nitrate than in animals receiving cyanide alone in this model despite significantly greater survival (p<0.01). The measured levels in these animals are also higher than levels in animals given high dose oral thiosulfate antidote (Groups 3 and 4). This is likely due to conversion of some blood hemoglobin to met-hemoglobin with greater carrying capacity for cyanide from the sodium nitrite administered with the intramuscular injection.

Plasma Thiocyanate Concentration: Plasma thiocyanate levels were higher in the sodium thiosulfate/sodium nitrite intramuscular treated animals as well (p<0.01), due to increased conversion of CN to thiocyanate, and similar to the oral thiosulfate treated animals (Groups 3 and 4).

## Discussion

This investigation was designed to study treatment for various scenarios of oral cyanide poisoning, including moderate and high dose ingestions. We focused on approaches that could be safely applied to individual or mass casualty settings, as well as to cases of suspected but unconfirmed exposures. All of the drugs we used are inexpensive and stable, have minimal side effects, and are FDA approved in some form (though not for this route, mode, dose or indication).

The 50 mg cyanide dose we used was very high, resulting in death in less than 15 min in Group 1 animals. Gastric alkalization by glycine could not be expected to prevent death, since all cyanide would eventually be absorbed, but we hypothesized that it could increase survival time. Thus, Group 2 animals that received glycine alone showed a trend toward longer survival, at 14 min compared to 10 min in Group 1 animals, but this difference was not statistically significant. Further studies would be required to determine whether higher glycine doses would further increase survival time, but this seems unlikely, since we confirmed that the gastric pH remained 9 in the animals that received glycine.

Group 3 animals that received sodium thiosulfate alone showed more signs of cyanide poisoning than Group 4 animals treated with sodium thiosulfate plus glycine, and although Group 3 animals slowly recovered, their base excess (reflecting lactate concentrations), and CWNIRS tissue hemoglobin oxygenation did not return to baseline. The gastric fluid of these animals developed a cloudy appearance, that we suspect is precipitated sulfur from acid instability of sodium thiosulfate [31]. This may be another contributing factor to the greater effectiveness of sodium thiosulfate when administered in alkaline conditions.

In contrast, Group 4 animals that received glycine and sodium thiosulfate showed rapid recovery and 100% survival. We hypothesize that gastric alkalization by glycine maintained the administered NaCN as CN- ion, rather than shifting to HCN gas as would occur in the acid milieu of the stomach. The hypothesis that gastric alkalization likely delayed systemic cyanide absorption, allowing sodium thiosulfate more time to neutralize cyanide, is supported by data in Figure 3 showing that blood cyanide concentrations increased at a slower rate in animals given glycine than in the control group.

Other possible mechanisms for the effectiveness of glycine and sodium thiosulfate include non-enzymatic conversion of cyanide to thiocyanate in the stomach at an elevated pH, or more systemic absorption of thiosulfate with enzymatic conversion of cyanide to thiocyanate. These possibilities were suggested by the more rapid and sustained rise in the plasma thiocyanate concentration in the glycine plus sodium thiosulfate-treated animals (Figure 3). To evaluate these two possible mechanisms, we measured thiocyanate in gastric fluid. Gastric samples obtained at baseline showed no significant thiocyanate. In Group 3 animals that received sodium thiosulfate and cyanide, a modest gradual increase in gastric thiocyanate was found. In contrast, in Group 4 animals that received glycine, cyanide, and sodium thiosulfate, the thiocyanate concentration increased rapidly. Thiocyanate is easily absorbed, and hence intra-gastric conversion of cyanide to thiocyanate likely occurred. However, some component of the oral thiosulfate effect appears to be due to systemic absorption and enzymatic conversion of cyanide to thiocyanate. This is evidenced by the relatively rapid rise in plasma thiocyanate in Group 3 animals (oral thiosulfate alone), despite low gastric thiocyanate concentrations at 10–20 min post ingestion and treatment (Figure 3 bottom and middle graphs).

Some of the administered glycine was likely absorbed, but it is unlikely that glycine absorption contributed to maintaining higher base excess concentrations in Group 4 animals since: 1) CWNIRS concurrently showed improved tissue hemoglobin oxygen curves, 2) the gastric thiocyanate concentrations were higher in Group 4 animals receiving glycine plus thiosulfate, and 3) Group 2 animals receiving glycine with cyanide showed rapid drop in base excess. Together, these findings support that stomach alkalization by glycine increased thiosulfate reaction with cyanide to generate thiocyanate in the stomach.

We used previously published methods [21,26,32–37] for applying near infrared technology to optically monitor the rate of cyanide poisoning and reversal by tracking tissue hemoglobin oxygenation. The CWNIRS data support the effectiveness of glycine and sodium thiosulfate for reversing metabolic parameters of cyanide poisoning in parallel with improvement in survival. In animals given glycine alone, tissue oxyhemoglobin concentrations quickly deteriorated as the animals began to succumb to cyanide toxicity. In contrast, animals given both sodium thiosulfate and glycine exhibited an initial rise in oxyhemoglobin and fall in deoxyhemoglobin due to inability of cyanide-poisoned cells to extract oxygen from hemoglobin. Subsequently, these animals recovered, and optical metabolic parameters returned toward baseline, due to effective neutralization of cyanide by sodium thiosulfate. Animals given cyanide alone or glycine plus cyanide demonstrated only a very brief rise in oxyhemoglobin, followed by precipitously decreased oxyhemoglobin associated with the rapid onset of terminal cardiovascular collapse.

Thus, the data show that gastric alkalization by glycine in conjunction with sodium thiosulfate appears to be effective in rescuing animals receiving supra-lethal doses of oral cyanide. Our *in-vitro* data showed that at high concentrations, non-enzymatic reaction between sodium thiosulfate and cyanide does occur, and is more rapid at alkaline pH. Chemical analysis of reaction rates between cyanide and sodium thiosulfate suggest the reaction can occur when the concentrations of cyanide and sodium thiosulfate are as high as those used in the current study (~40 mM cyanide and 0.75 M sodium thiosulfate) [14,30]. Reaction rates are expected to be faster at alkaline pH, than at acid pH [14,30], as we observed *in-vitro* and *in-vivo*.

Several important limitations of this study will need to be addressed in subsequent animal studies before considering oral glycine and sodium thiosulfate as antidotes for oral cyanide poisoning. Most importantly, rabbits have a very rapid metabolic rate compared to humans and we administered an extremely high dose of cyanide (50 mg), which is close to a lethal dose for a full grown adult human. Thus, the therapeutic time-window for antidote administration was extremely short, and we administered the glycine and sodium thiosulfate concurrently with the oral cyanide in the 50 mg dose animal groups. A combination of initial systemic (intravenous or intramuscular) antidote followed by oral antidote may be preferable for acute oral cyanide ingestion, particularly in high dose symptomatic cases. In the lower dose, 25 mg oral cyanide exposed animals, improved overall survival (71%) was achieved with delayed intramuscular antidote injection alone group animals, but 29% still expired. In the severely poisoned (50 mg cyanide ingestion group where apnea occurs between 1 and 3.5 min without treatment), delayed treatment animals receiving combined intramuscular and oral antidote had increased survival time compared to controls, and 29% of the animals were able to survive. The systemic (intramuscular) antidote is available very rapidly neutralize absorbed cyanide, while the oral antidote is used neutralize the gastrointestinal reservoir. Such combination therapy approaches appear feasible based on our studies. Large animal models, with metabolic rates more analogous to adult humans and consequently, a longer potential therapeutic time window, will need to be studied using longer delays in antidote administration that more closely parallel treatable human exposures. Second, the effects of food in the stomach and GI tract on the response to treatment need to be investigated. Food delays cyanide absorption [38, 39], but could affect the ability of glycine to raise pH, and could possibly interfere with the ability of sodium thiosulfate to neutralize cyanide. Additionally, in apneic patients, oral antidote alone would not likely be a clinical consideration, because it would not be effective quickly enough and cannot be administered until the airway has been secured and a nasogastric tube placed.

Therefore, we speculate that oral antidote administration in mass casualty situations may be limited to known or possible ingestion victims who are alert enough to swallow safely, while in severe symptomatic ingestion cases, enteral administration (via nasogastric tube) will likely only be administrable once airway protection has been secured.

In the future, other reactive sulfur containing compounds with higher reported cyanide reaction rate constants with cyanide [14,30] could be considered for further investigation. An important question of whether chronic cyanide toxicity associated with cyanogenic foods could be mitigated by repeated glycine and sodium thiosulfate ingestion is complex and will require careful study. It is conceivable that gastric alkalization could paradoxically lead to exacerbation of the ingestion from greater conversion of cyanide through the intestine due if increased passage of cyanogenic compounds through stomach occurs under alkaline conditions.

The FDA recognizes that antidotes for cyanide poisoning cannot be tested in humans and requires that efficacy be determined in at least two different animal species (preferably non-rodent), under GLP conditions. This study only observed short-term survival, and long-term effects will also require further investigation.

## Conclusions

In conclusion, these studies indicate that the combination of oral glycine and sodium thiosulfate appears to have potential for treating high dose acute cyanide ingestion and merits further investigation. The combination of systemic and oral therapy may provide additional options. The antidote candidates used in this study are safe, inexpensive, and have been FDA approved for other indications.

## Abbreviations

CWNIRS: Continuous Wave Near Infrared Spectroscopy
OxyHb: Oxyhemoglobin
DeoxyHb: Deoxyhemoglobin
